# A five-year retrospective review of snakebite patients admitted to a tertiary university hospital in Malaysia

**DOI:** 10.1186/1865-1380-4-41

**Published:** 2011-07-13

**Authors:** Keng Sheng Chew, Heng Wei Khor, Rashidi Ahmad, Nik Hisamuddin Nik Abdul Rahman

**Affiliations:** 1 Emergency Medicine Department, School of Medical Sciences, 16150 Kubang Kerian, Universiti Sains Malaysia, Penang, Malaysia; 2 Advanced Medical and Dental Institute, Universiti Sains Malaysia, No 1-8 (Lot 8), Persiaran Seksyen 4/1, Bandar Putra Bertam, 13200 Kepala Batas, Pulau Pinang, Malaysia

**Keywords:** snake bites, envenomation, antivenoms

## Abstract

**Background:**

Although the majority of the snakebite cases in Malaysia are due to non-venomous snakes, venomous bites cause significant morbidity and mortality if treatment measures, especially ant-venom therapy, are delayed.

**Methods:**

To determine the demographic characteristics, we conducted a retrospective study on all snakebite patients admitted to the Emergency Department of Hospital Universiti Sains Malaysia (HUSM) from January 2006 to December 2010.

**Results:**

In the majority of the 260 cases that we found (138 cases or 52.9%), the snake species was unidentified. The most common venomous snakebites among the identified species were caused by cobras (52 cases or 20%). Cobra bites are significantly more likely to result in severe envenomation compared to non-cobra bites. Post hoc analysis also showed that cobra bite patients are significantly less likely to have complete recovery than non-cobra bite patients (48 cases, 75.0% vs. 53 cases, 94.6%; *p *= 0.003) and more likely to result in local gangrene (11 cases, 17.2% vs. 3 cases, 5.4%; *p *= 0.044).

**Conclusion:**

Cobra bites are significantly more likely to result in severe envenomation needing anti-venom administration and more likely to result in local gangrene, and the patients are significantly less likely to have complete recovery than those with non-cobra bites.

## Introduction

As early as 1963, it was shown that the majority (74.0%) of snakebite incidents in Malaysia occurred in the four northern states of Peninsular Malaysia [1]. Fortunately, most snakes in Malaysia are non-venomous and are relatively harmless to humans. Only about 17 out of the 105 strict land snakes in Malaysia are venomous [2].

In fact, even bites of venomous snakes are often not life threatening for humans unless a sufficient amount of venom is injected at the time of the bite. In fact, most bites are dry bites because they are defensive [1]. Nonetheless, while this may be true, the more challenging problem is accurate species identification [3] by the witnesses. It is often difficult to even identify whether a snake is venomous or not, let alone identify its exact species [2].

Venomous snakes in Malaysia can be divided into three main groups - two groups of land snakes and one of sea snakes. The two main groups of land snakes are the Elipidae (such as cobras) and the Viperidae (i.e., pit vipers). All 22 species of sea snakes in Malaysia are considered venomous [2]. As the habitat of most sea snakes is along the shallow coastal waters, fishermen are the the group considered most at risk for sea snake bites [2]. Interestingly, sea snake bites cause little or no pain or edema at the site of the bite [2].

Myotoxicity is venom toxicity that results in myotoxic effects such as muscular pain, stiffness and myoglobinuria. Myoglobinuria is characterized by the brown discoloration of urine and usually eventual respiratory failure. Neurotoxicity is defined as a toxicity that results in neurotoxic effects such as muscular weakness, spreading paralysis (within 15 min to 2 h), dysphagia, dysphasia, ptosis, external opthalmoplegia as well as slowed, labored breathing, culminating in respiratory arrest with or without convulsions. Hemotoxicity results in hemotoxic effects such as echymoses, petechial hemorrhage, epistaxis, hematemesis, malena, coagulopathy, hematuria or any bleeding manifestations not attributable to other causes. The venom of pit vipers often results predominantly in hemotoxicity, the venom of Elapidae predominantly in neurotoxicity, whereas that of sea snakes predominantly causes myotoxicity [2], although there are often overlaps in symptom presentation.

The purpose of this study is to map out the demographic characteristics, clinical profiles and manifestations, and the outcomes for snakebite patients admitted to our hospital over the last 5 years.

### Methodology

This is a retrospective study involving all snakebite patients admitted to the Emergency Department of Hospital Universiti Sains Malaysia (HUSM) from January 2006 to December 2010.

After retrieving the registration numbers and case notes for all snakebite patients admitted to HUSM during the stipulated time, we reviewed all the relevant data needed for our analysis. Besides demographic data, the analyzed variables included the type of snake, severity of envenomation, time periods where the bites occurred, common symptoms suggestive of hemotoxicity, myotoxicity and neurotoxicity, local symptoms including conditions of wounds and recovery progress.

Cases where the patients were 'discharged against medical advice' were excluded. Cases of 'unknown' bites in the absence of fang marks or any other symptoms suggestive of venomous snakebites were also excluded. This study was conducted with the approval of our institutional research ethics board from the Advanced Dental and Medical Institute, Universiti Sains Malaysia. Permission was similarly obtained from the Hospital Director to allow us to access the information from the patients' case notes strictly for the purpose of this research.

Mild envenomation is defined as minimal or mild swelling of a less than 4 cm increase in limb circumference with no clinical evidence of local gangrene or systemic symptoms. Moderate envenomation is defined as resulting in local swelling of 4 cm or more and/or showing clinical evidence of local gangrene with minimal or no systemic symptoms. Severe envenomation results in clinical evidence of systemic poisoning that potentially can be fatal [4].

Statistical analysis was done using the Statistical Package for Social Sciences (SPSS) version 18 for Windows. Comparisons of categorical data were carried out using Pearson's chi-square or Fisher's exact test where appropriate. A *p *value of less than 0.05 was taken as statistically significant.

## Results

A total of 260 snakebite patients were analyzed in the 5-year period from January 2006 to December 2010. Of these 260 cases, 64 (24.5%) were cobra bites, 52 (20.0%) viper bites, 4 sea snake bites (1.5%), 3 python bites (1.1%) and 138 unknown snakebites (52.9%).

**Figure 1:**
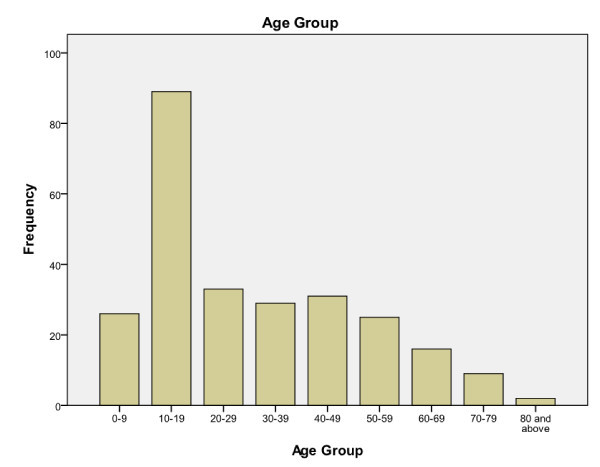
**Frequency of snakebite cases according to age groups**.

**Figure 2:**
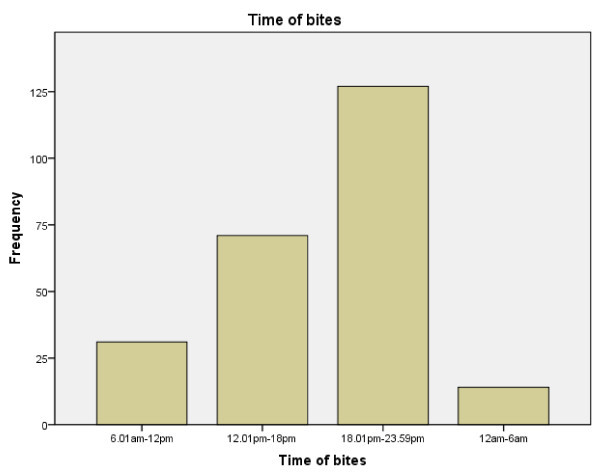
**Number of snakebite cases according to time period**.

In terms of the patients' age groups, the highest number of cases (89 cases or 34.2%) occurred in the 10-19-year-old category (Figure 1). The youngest victim was 4 years old, and the oldest snakebite patient was 88 years old. The number of male patients was higher than the number of female patients [154 (59.2%) versus 106 (40.8%)]. A total of 61 (23.5%) snakebites occurred during indoor activities and 118 (45.4%) during outdoor activities (81 cases with missing data). Most of the snakebites occurred during the 6-h evening period from 1800 to 2359 hours (Figure 2).

The mean of total admission days was 3.90 (SD ± 5.14) days. The longest hospital stay was 40 days. Six out of 260 patients (2.31%) were admitted to the intensive care unit (ICU). These six patients all had severe envenomation, and two were mechanically ventilated.

Sixty patients (23.1%) presented with symptoms suggestive of myotoxicity, 9 (3.5%) with symptoms suggestive of hemotoxicity and 35 (13.5%) with symptoms suggestive of neurotoxicity. Nine patients (3.5%) presented with overlapping features of both neurotoxicity and myotoxicity, but not hemotoxicity. Six patients (2.31%) presented with overlapping features suggestive of both myotoxicity and hemotoxicity, but none of the patients presented with symptoms of both hemotoxicity and neurotoxicity. Regarding the bite sites, 191 patients (73.45%) were bitten on the lower limbs, whereas 60 (23.10%) were bitten on the upper limbs (9 patients or 3.45% with missing data). Although 98 patients (37.7%) presented with signs and symptoms suggestive of severe envenomation, only 48 (18.5%) received anti-venom. The details of the common symptoms experienced by the patients are presented in Table 1.

**Table 1:** Symptoms experienced by the patients clustered according to the different types of venom toxicities

Symptoms	Frequency (*n *= 260)	Percentage (%)
***Symptoms suggestive of myotoxicity***		
Muscle tenderness	59	22.7
Myoglobinuria	2	0.8
		
***Symptoms suggestive of hemotoxicity***		
Coagulopathy	6	2.3
Hematemesis	1	0.4
Hematuria	1	0.4
Thrombocytopenia	3	1.2
		
***Symptoms suggestive of neurotoxicity***		
Numbness	1	0.4
Ventilated	3	1.2
"Heavy" eyelids/ptosis	19	7.3
Paralysis of facial muscles	1	0.4
Difficulty in swallowing secretions	1	0.4
Respiratory failure	22	8.5

Among the 120 identified venomous snakes, 64 were cobras and 56 non-cobras. Of the 64 cobra-bite patients, 42 (65.6%) had severe envenomation compared to only 15 (26.8%) such cases in the non-cobra-bite group (*p *< 0.001, chi-square test applied) (Tables 2 and 3). In a similar vein, more patients bitten by cobras received anti-venom than patients bitten by other snakes (21 cases, 32.8% vs. 12 cases, 21.4% respectively), although this difference did not reach statistical significance (*p *= 0.164) (Table 3).

**Table 2:** Types of snake species and grading of envenomation

Snake types	Clinical severity				Total
	Minimal	Moderate	Severe	None	
Cobra	16	5	42	1	64
Viper	14	18	14	6	52
Sea snake	1	2	1	0	4
Unknown	46	44	40	7	136
Total	78	70	98	14	260

**Table 3:** Results of post-hoc analysis of comparison between cobra and non-cobra bites

	Cobra	Non-cobra	*p *value**
Results in severe envenomation	42 (65.6%)	15 (26.8%)	< 0.001
Requiring anti-venom administration	21 (32.8%)	12 (21.4%)	0.164
Bitten at lower limb more than upper limb	45(57.7%)	33 (42.3%)	0.290
Results in neurotoxicity	18 (28.1%)	1 (1.8%)	< 0.001
Complete recovery	48 (75.0%)	53 (94.6%)	0.003
Complicated with gangrene in at least part of the bite site	11 (17.2%)	3 (5.4%)	0.044

** All analysis done using Pearson's chi-square test.

In term of local effects, fang marks were noted in 186 patients (71.5%) and gangrene in 17 (6.5%). Six patients (2.3%) had clinical features suggestive of compartment syndrome, and one eventually underwent fasciotomy. Furthermore, 13 patients (5.0%) developed secondary infections (Table 4).

**Table 4:** Local symptoms experienced by victims

General	Frequency (*n *= 260)	Percentage (%)
Fang marks	186	71.5
Blistering	14	5.4
Gangrene	17	6.5
Cellulitis	22	8.5
Skin discoloration	33	12.7
Compartment syndrome	6	2.3
Infected wounds	13	5.0

Up to 24% of the patients had a time lapse of between 1 to 4 h before presenting to the hospital (Table 5). Approximately 44% of our patients were referred from district hospitals (Table 5).

**Table 5:** Time lapsed before presentation to the hospital

Time interval (in hours)	Number of cases (n = 212)*	Percentage (%)
Less than 1 h	49	18.8
1 - 4 h	72	27.7
4 - 24 h	6	2.3
More than 1 day	3	1.2
Referred case	120	46.2
Total	250	96.2

*Missing data in 10 cases (3.8%)

## Discussion

In this study, we found that in the majority of snakebite cases (52.9%), the exact snake species was not identified, although in these unidentified cases fang marks or other symptoms suggestive of venomous bites were present. This is not surprising given the fact that these were often quick, defensive bites [1]. The patients were frequently anxious and frightened, which often could cloud their ability to identify the species even among those patients who had some knowledge of the appearances of the different common snake species.

Most earlier epidemiological studies done in the 1960s to 1990s showed that majority of venomous bites were due to pit vipers [1,5-7]. However, our findings, as well as the more recent studies done from the 1990s onwards, show a possible changing trend with cobra bites being the more common type compared to pit viper bites [2,8,9]. Although the reasons behind this trend could not be ascertained, one of the possibilities postulated by Jamaiah et al. (2006) was the rapid and intense land development for housing and industrial projects. Such urbanization has inadvertently resulted in humans encroaching into the natural habitats of these creatures [8].

Furthermore, contrary to what many people may believe, the cobra is actually not an aggressive snake and avoids encountering humans as much as possible [1,8]. It only attacks when provoked or accidentally stepped on. If cornered, however, the king cobra can be extremely dangerous because of the large amount of venom it is capable of delivering in a bite [2].

Not only did we find that cobra bites made up the majority of the identified venomous snakebites in our study, but cobra bites were more likely to result in severe envenomation compared to other species. Post-hoc analysis also showed that cobra bites were more statistically likely to cause local gangrene at bite sites than non-cobra bites, and the patients were less statistically likely to achieve complete recovery. This may be due to the fact that the venom of cobras, or the Elapidae as a whole, often results in neurotoxicity [1-3].

The observation that bites on the lower limbs were three times as common than bites on the upper limbs suggests that in most cases the snake was stepped on inadvertently [1,7]. Most of the new patients had a time lapse of between 1 and 4 h before presentation to the hospital. This trend does not differ significantly from that found in a study done by Reid et al. in 1963 [1]. In view of the great importance of anti-venom particularly in cases of moderate to severe envenomation, greater emphasis should be placed on patient education. One of the reasons for the delay before hospital presentation mentioned by Reid et al. (1963) was that the community preferred trying traditional and folk medicine first rather than coming to the hospital immediately [1].

There are a number of limitations in our study. Our data on the species of snakes taken from the hospital case notes were based entirely on the description given by the patients and other witnesses. Unlike some other studies, we were reluctant to categorize our data on 'type of snake' into suspected cases and confirmed cases, because we found this categorization to be rather arbitrary since there was no herpetologist in our center to help us with this task. Furthermore, the many confusing and missing data in the case notes render such categorization difficult. This study was conducted only in one center in Malaysia over a 5-year period, and therefore, the epidemiological findings may not truly reflect the epidemiological trend in Malaysia as a whole. Future multicenter studies should be conducted to validate these findings.

## Conclusion

Overall, from this study, we found that in more than 50% of the snakebite cases admitted to HUSM from 2006 to 2010, the species of snake was not identified. Among those identified, the most common venomous snakebites were cobra bites. Cobra bites are significantly more likely to result in severe envenomation needing anti-venom administration. Post-hoc analysis also showed that patients with cobra bites were significantly less likely to achieve complete recovery than those with non-cobra bites and more likely to develop local gangrene.

## Patient's consent

No direct consent was taken from the patients as this is a retrospective study. Details of the history, clinical findings, admissions and outcomes were obtained from the hospital records. Consent, instead, was obtained from the Hospital Director to use the information contained in the patient record solely for the educational purpose of this research only.

## Competing interests

The authors declare that they have no competing interests.

## Authors' contributions

KSC contributed in data collection, results analysis and was directly involved in writing this manuscript. HWK contributed in the initial conception, drawing up the study design, data collection as well as analysis of this study RA contributed in the initial conception and designing the methodology of this study. NHNAR contributed in the study design and result analysis of this study.
